# Factors Associated With Nonadherence to Hypertension Treatment Among Young Adults in Korea

**DOI:** 10.1155/ijhy/4840707

**Published:** 2025-10-14

**Authors:** Yoon Hee Cho, Joohyun Lee

**Affiliations:** ^1^Department of Nursing, College of Nursing, Dankook University, Cheonan 31116, Republic of Korea; ^2^Department of Nursing, College of Nursing, Eulji University, Seongnam 13135, Republic of Korea

**Keywords:** disease management, hypertension, KCHS, young adults

## Abstract

This study investigated hypertension management in young adults in Korea using data from the 2023 Korean Community Health Survey. We examined treatment patterns and identified factors associated with untreated cases among adults aged 20–39 years diagnosed with hypertension. Among young adults, 21.2% were receiving both drug treatment and lifestyle modification, while 28.6% were neither receiving drug treatment nor lifestyle modification. In addition, 35.5% were receiving only drug treatment, and 14.8% were practicing only lifestyle modification. The percentage of young adults who were not practicing both drug treatment and lifestyle modification was higher than that of adults aged 40 or older. These were associated with male sex (OR: 1.427, 95% CI: 1.12–1.82), BMI (OR: 0.932, 95% CI: 0.91–0.95), current smoking (OR: 1.366, 95% CI: 1.06–1.77), alcohol consumption (≤ once/week, OR: 1.654, 95% CI: 1.14–2.39; ≥ twice/week, OR: 2.484, 95% CI: 1.59–3.87), awareness of one's own blood pressure (OR: 0.194, 95% CI: 0.15–0.26), knowledge of myocardial infarction symptoms (OR: 0.779, 95% CI: 0.71–0.86), education about hypertension management (OR: 0.648, 95% CI: 0.52–0.80), and social network level (OR: 0.934, 95% CI: 0.91–0.96). These findings highlight the need for tailored interventions to improve hypertension awareness and management in young adults.

## 1. Introduction

Hypertension is a major global public health issue, recognized as a serious medical condition that increases morbidity and mortality from cardiovascular diseases and strokes and even cancers [1, 2]. According to the 2023 Korean Hypertension Fact Sheet, 28.0% of adults aged 20 and older in Korea have been reported to have hypertension [3]. Although hypertension predominantly occurs in older adults [1], hypertension in young adults is also a significant concern due to the increased risk of long-term complications and target organ damage [4].

Globally, the prevalence of elevated blood pressure or hypertension among young people, including adolescents, has been steadily rising [5, 6]. This trend is similar in Korea, where recent data from the Health Insurance Review and Assessment Service indicate that, over the past 5 years, the rate of hypertension diagnoses has increased most significantly among adults in their twenties and thirties compared to other age groups [7]. Known risk factors for hypertension include unhealthy diets, lack of physical activity, tobacco and alcohol use, and obesity [1]. In young adults, hypertension is more prevalent among males than females and is often associated with factors such as high tobacco and alcohol consumption, being overweight or obese, and experiencing psychological stress [1, 8].

Early intervention in the management of hypertension among young adults, through appropriate medication and lifestyle modifications, is essential to reduce the future risk of complications, including cardiovascular diseases [1, 9, 10]. This includes both pharmacological treatment and nonpharmacological approaches such as reducing salt intake, increasing the consumption of fruits and vegetables, engaging in regular physical activity, avoiding tobacco use, reducing alcohol consumption, and maintaining a healthy weight [1]. However, previous studies have indicated that young adults with hypertension tend to have lower awareness of their blood pressure, slower diagnosis, and poorer blood pressure control compared to older adults [5]. Moreover, young adults may underestimate the adverse effects of hypertension or fail to recognize their condition [8].

A survey in Korea reported low awareness (35.8%), treatment (30.9%), and control rates (23.0%) among hypertensive adults aged 30 to 49 [11]. Additionally, only 12.5% of diagnosed hypertensive patients reported having received education on hypertension management [12]. Hypertension education is a crucial strategy for improving lifestyle habits and promoting self-management among young adults with hypertension [12]. Thus, assessing the awareness and treatment rates of hypertension, along with the factors influencing these rates, is essential for developing tailored interventions for young adults. In particular, it is necessary to identify the reasons why medication or lifestyle modification is not implemented even when suggested.

This study utilized nationwide data from Korea to examine the treatment status and geographic distribution of young adults diagnosed with hypertension and to identify factors associated with nonadherence to hypertension management methods.

## 2. Methods

### 2.1. Research Design

This study is a secondary analysis designed to investigate the national distribution and adherence to treatment among young adults diagnosed with hypertension and to identify factors associated with nonadherence to hypertension management methods. The research data were derived from the raw data of the Korean Community Health Survey (KCHS) conducted in 2023. The KCHS is an annual survey administered by public health centers to support the establishment and evaluation of community-specific health policies in Korea [13].

### 2.2. Participants

The participants in this study were adults aged 20 to 39 who participated in the 2023 KCHS and had been previously diagnosed with hypertension by a physician. The KCHS is an interview survey conducted annually by all public health centers nationwide and was conducted on approximately 900 adults per health center. A total of approximately 230 thousand adults participated nationwide in 2023. This study targeted 1484 of 44,236 adults aged 20–39 who participated in the 2023 survey and were diagnosed with hypertension and answered all questions about hypertension management methods. Participants were divided into four groups based on their current blood pressure management methods: (1) a group that took medications and improved lifestyle habits (exercise or diet), (2) a group that only improved lifestyle habits (exercise or dietary adjustments), (3) a group that only took medications, and (4) a group that did not take medications or improve lifestyle habits at all.

### 2.3. Data Collection and Research Ethics

The KCHS is a nationwide health survey conducted annually by local health centers under the Korea Disease Control and Prevention Agency. The survey targets adults residing in selected households, with trained interviewers conducting face-to-face interviews [13]. The researchers accessed the 2023 raw data in compliance with the data usage guidelines after submitting a data usage pledge and research proposal for approval. All data were provided in an anonymized format, with restricted access to the research team. This study was approved by the Institutional Review Board (IRB) of the researchers' affiliated institution (IRB No. EUIRB2024-110), which waived the requirement for further ethical review.

### 2.4. Measurements

#### 2.4.1. Treatment Status After Hypertension Diagnosis

Participants were classified based on their current management of hypertension: (1) medications and lifestyle modifications, (2) lifestyle modifications only (exercise or dietary adjustments), (3) medications only, and (4) nonadherence to treatments (a group that did not take medications or improve lifestyle habits at all). This classification considered whether participants had been diagnosed with hypertension by a physician and whether they were currently managing it through medication, exercise, or diet. First, participants answered “yes” or “no” to the question, “Have you ever been diagnosed with high blood pressure by a doctor?” Those who were diagnosed with high blood pressure were asked additional questions about their treatment of high blood pressure. The questions were, “Are you currently exercising or dieting to treat your high blood pressure?” and “Are you currently taking blood pressure medication?”

#### 2.4.2. Hypertension-Related Characteristics

Variables associated with hypertension included sex, body mass index (BMI), smoking behavior, alcohol consumption, awareness of personal blood pressure, knowledge of stroke and myocardial infarction symptoms, stress levels, addiction experiences, depressive experiences, education on hypertension management, and social network level. BMI was calculated from self-reported height and weight. Smoking status was categorized as nonsmoker, former smoker, or current smoker, based on questions regarding the use of tobacco products, including e-cigarettes. Alcohol consumption was classified based on drinking frequency over the past year: nondrinker, drinking once per week or less, and drinking more than twice per week. Blood pressure awareness was assessed by whether participants knew their blood pressure readings. Hypertension education was determined by whether participants had received information on blood pressure management following diagnosis. Knowledge of stroke and myocardial infarction symptoms was assessed using five questions for each condition, scored on a scale of 0–5, with incorrect or “do not know” responses receiving a score of 0. Addiction experience was assessed by determining whether excessive use of the internet, gaming, smartphones, or gambling had impacted daily life within the past year. Depressive experience was assessed by determining whether feelings of sadness and despair had interfered with daily life for more than 2 weeks over the past year, and stress level was measured on a 4-point scale, ranging from feeling very little to feeling very much. Social network level was scored from 1 to 6 for each item related to the frequency of contact with relatives, neighbors, and friends, resulting in a maximum score of 18.

### 2.5. Statistical Analyses

The KCHS, being a sample survey, incorporates stratification, clustering, and weighting variables, which should be accounted for in the analysis to reflect the complex sample design [13]. In this study, analysis was conducted in accordance with the recommendations of the data usage guidelines [13]. The treatment status of young adults diagnosed with hypertension was compared to that of adults aged 40 and above using unweighted frequency and weighted percentages. The geographic distribution of treatment status among young adults was visualized through the Statistical Geographic Information Service Plus, an open-source platform [14]. Group characteristics based on treatment status were presented with unweighted frequencies, weighted percentages, means, and standard errors. Factors associated with treatment status were analyzed using multinomial logistic regression, with odds ratios and 95% confidence intervals provided. All analyses were conducted using SPSS version 28.0 (IBM, Armonk, NY, USA).

## 3. Results

### 3.1. Treatment of Hypertension After Diagnosis in Young Adults

**Figure 1:**
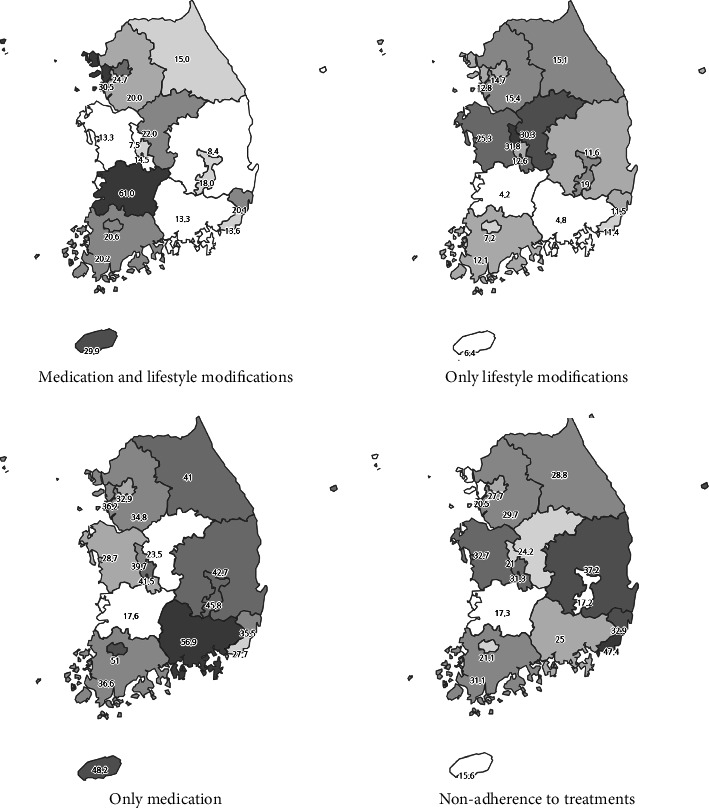
National distribution of treatment rates after diagnosis of hypertension in young adults.

Table 1 presents a comparison of hypertension treatment status after diagnosis among young adults (20–30 s) in Korea with those aged 40 and older. Among young adults, 21.2% engaged in both medications and lifestyle adjustments, 35.5% relied solely on medications, 14.8% adopted only lifestyle modifications, and 28.6% did not use any medication or lifestyle modifications at all. The proportion of those not using any medication or lifestyle modifications was notably higher compared to 2.7% among adults over 40.  Figure 1 illustrates the treatment status by region among young adults, showing distinct regional variations in treatment approaches. The proportion of untreated people varied by region, ranging from 47.5% to 17.2%.

### 3.2. Characteristics of Groups by Treatment Status After Diagnosis of Hypertension

Table 2 provides characteristics of young adults diagnosed with hypertension and the differences across groups. Among the young adults diagnosed with hypertension, 81.3% were male, with an average BMI of 28.24. Current smoking and alcohol consumption (two or more times per week) rates were 34.6% and 26.4%, respectively. Awareness of blood pressure status was reported by 88.1%, while knowledge of stroke and myocardial infarction symptoms averaged 3.74 and 3.53 on a 5-point scale. Reported stress levels were 2.68 out of 4, with addiction symptoms in 26.4% and depressive symptoms in 9.5% of respondents. The rate of hypertension management education was 34.0%, and social network levels averaged 9.39 out of 18.

Analyzing characteristics by group, significant differences were noted across all variables. In comparing Group A (medications and lifestyle adjustments) with Group D (nonadherence to treatments), a higher percentage of men was found in Group D (83.7%) than in Group A (77.2%). Group D also reported higher rates of current smoking (35.5% vs. 26.5%) and frequent alcohol consumption (29.7% vs. 18.4%). Awareness of blood pressure was lower in Group D (77.5%) than in Group A (95.1%), as was knowledge of stroke and myocardial infarction symptoms (3.31 and 3.06 in Group D vs. 4.13 and 3.92 in Group A). Group D had higher rates of addiction (34.7%) and depression (29.9%) and lower rates of hypertension management education (28.7% vs. 42.9%).

### 3.3. Associated Factors in the Group Without Treatment After Diagnosis of Hypertension

Factors associated with nonadherence to treatment among young adults with hypertension, relative to those engaging in both medication and lifestyle management, are summarized in Table 3. Key factors included sex (OR: 1.427, 95% CI: 1.12–1.82), BMI (OR: 0.932, 95% CI: 0.91–0.95), current smoking (OR: 1.366, 95% CI: 1.06–1.77), and alcohol consumption (≤ once/week, OR: 1.654, 95% CI: 1.14–2.39; ≥ twice/week, OR: 2.484, 95% CI: 1.59–3.87). Additional factors included awareness of one's blood pressure (OR: 0.194, 95% CI: 0.15–0.26), knowledge of myocardial infarction symptoms (OR: 0.779, 95% CI: 0.71–0.86), experiences of addiction (OR: 1.630, 95% CI: 1.27–2.09), prior education on hypertension management (OR: 0.648, 95% CI: 0.52–0.80), and social network level (OR: 0.934, 95% CI: 0.91–0.96).

## 4. Discussion

This study examined the treatment status and national distribution of young adults diagnosed with hypertension using data from the KCHS. The results showed that 28.6% of young adults with diagnosed hypertension in Korea were not taking any medication or making any lifestyle modifications, which was higher than the rate of adults aged 40 years or older. Although age differences exist, previous studies have estimated a hypertension prevalence of approximately 15% among adults aged 30–49 in Korea [11], underscoring that many young adults remain untreated despite high blood pressure levels. According to a recent hypertension fact sheet by The Korean Society of Hypertension, approximately 890,000 young adults in their 20 s and 30 s have hypertension, with low rates of awareness and treatment contributing to an overall increase in hypertension cases in Korea [15]. In this survey, 1484 (3.4%) of 44,236 adults aged 20 to 39 responded that they had been diagnosed with high blood pressure. This figure is lower than previously reported, but considering the low awareness of high blood pressure among young adults, it is estimated that more young adults have high blood pressure. The long-term implications of uncontrolled hypertension in young adults include various complications and impacts on quality of life [4], emphasizing the need for targeted interventions to improve treatment rates in this population.

The study also found that the treatment rate after hypertension diagnosis varied widely by region, with treatment nonadherence rates ranging from 17.2% to 47.5%. Workers at public health centers should assess treatment status by region and identify underlying causes in areas with lower treatment rates.

Logistic regression analysis comparing individuals who were nonadherent to treatment with those who practiced both medications and lifestyle modifications showed that men were more likely to be nonadherent (OR: 1.427, 95% CI: 1.12–1.82), consistent with previous studies on gender differences in hypertension treatment and management among young adults [16]. Healthcare providers should consider these differences when developing appropriate intervention plans to improve treatment and care for both men and women, and additional efforts are needed to understand nonadherence in men in particular.

Smoking and alcohol consumption are well-known factors associated with hypertension prevalence [1]. Our study also showed that current smokers (OR: 1.366, 95% CI: 1.06–1.77) and frequent drinkers (OR: 2.484, 95% CI: 1.59–3.87) were more likely to be untreated, highlighting the need for lifestyle interventions alongside medical treatment for effective hypertension management.

Previous studies have shown that young adults have lower awareness of their blood pressure and often experience delayed diagnosis compared to older patients [5]. Many young adults also underestimate the adverse effects of hypertension or are reluctant to acknowledge their condition [8]. Consistent with this, our findings indicate that untreated young adults were often unaware of their blood pressure (OR: 0.194, 95% CI: 0.15–0.26) and had limited knowledge of myocardial infarction as a hypertension complication (OR: 0.779, 95% CI: 0.71–0.86). Additionally, those who had not received education on hypertension management (OR: 0.648, 95% CI: 0.52–0.80) were more likely to be untreated. Therefore, health providers should actively educate young adults diagnosed with hypertension about management strategies and complications, ensure they know their blood pressure levels, and encourage regular monitoring at health centers or hospitals.

Although this study relied on secondary analysis and could not include direct clinical verification of hypertension diagnoses, previous studies have shown little discrepancy between self-reported and medical records of blood pressure measurements in young adults [17]. Given the high treatment nonadherence rate observed in this study, these findings offer important implications for public health in Korea. Future studies should propose strategies to improve treatment rates among young adults with hypertension.

This study has some limitations. First, as a secondary analysis, it could not directly confirm all aspects of hypertension diagnosis and treatment. Future research should directly measure blood pressure in young adults and more precisely assess their treatment status. Second, as a cross-sectional study, it cannot establish causal relationships between treatment behaviors and associated factors, warranting caution in interpreting related factors.

## 5. Conclusions

This study used 2023 KCHS data to examine the treatment status and national distribution of young adults diagnosed with hypertension. Among young adults with hypertension in Korea, 28.6% were found to be untreated, with treatment rates varying across regions. Compared to individuals receiving both pharmacological and nonpharmacological treatment, untreated individuals showed associations with gender, BMI, smoking, alcohol consumption, history of substance use, awareness of their blood pressure, knowledge of myocardial infarction, education on hypertension management, and social network level. These findings suggest the need for early, proactive interventions to increase hypertension treatment rates among young adults in Korea.

## Figures and Tables

**Table 1:** Treatment status after diagnosis of hypertension in adults.

	Young adults		Adults over 40 years old	
	Unweighted frequency (weighted frequency %)			
Medication and lifestyle modifications	294	(21.2)	16,009	(27.9)
Only lifestyle modification	20	(14.8)	751	(1.7)
Only medication	582	(35.5)	52,751	(67.7)
Nonadherence to treatments	388	(28.6)	1586	(2.7)
Total	1484	(100.0)	71,097	(100.0)

**Table 2:** Characteristics of groups by treatment status after diagnosis of hypertension in young adults.

Variables	Total	Group A	Group B	Group C	Group D	*p*
	*M* ± SE/unweighted frequency (weighted frequency %)					
Sex (man)	1162 (81.3)	218 (77.2)	186 (88.0)	436 (79.0)	322 (83.7)	< 0.001
BMI	28.24 ± 0.07	28.97 ± 0.15	27.42 ± 0.17	28.74 ± 0.13	27.49 ± 0.16	< 0.001
Smoking (nonsmoking)	637 (45.0)	149 (49.6)	96 (45.3)	259 (42.0)	169 (45.1)	< 0.001
Smoking (past smoking)	294 (20.4)	67 (23.9)	38 (14.0)	118 (21.8)	71 (19.4)	
Smoking (current smoking)	517 (34.6)	78 (26.5)	86 (40.7)	205 (36.3)	148 (35.5)	
Alcohol drinking (nondrinking)	235 (13.1)	50 (16.3)	30 (9.8)	114 (16.0)	41 (8.8)	< 0.001
Alcohol drinking (≤ once/week)	860 (60.5)	185 (65.2)	132 (65.7)	308 (54.8)	235 (61.5)	
Alcohol drinking (≥ twice/week)	389 (26.4)	59 (18.4)	58 (24.5)	160 (29.2)	112 (29.7)	
Being aware of one's own blood pressure (yes)	1317 (88.1)	281 (95.1)	199 (88.5)	537 (91.9)	300 (77.5)	< 0.001
Knowledge of stroke warning signs	3.74 ± 0.02	4.13 ± 0.36	3.61 ± 0.06	3.88 ± 0.04	3.31 ± 0.05	< 0.001
Knowledge of myocardial infarction signs	3.53 ± 0.02	3.92 ± 0.04	3.48 ± 0.05	3.71 ± 0.04	3.06 ± 0.04	< 0.001
Stress level	2.68 ± 0.01	2.73 ± 0.02	2.73 ± 0.02	2.65 ± 0.0	2.67 ± 0.03	0.009
Addiction experience (yes)	364 (26.4)	65 (25.2)	59 (25.8)	113 (20.6)	127 (34.7)	< 0.001
Depressive experience (yes)	131 (9.5)	26 (23.3)	20 (17.3)	48 (29.5)	37 (29.9)	0.014
Education on how to manage hypertension (yes)	496 (34.0)	118 (42.9)	94 (40.8)	173 (30.1)	111 (28.7)	< 0.001
Social network level	9.39 ± 0.05	9.84 ± 0.08	9.27 ± 0.10	9.49 ± 0.08	8.98 ± 0.11	< 0.001

*Note:* Group A: those undertaking both medication and lifestyle modifications (exercise and dietary adjustments); Group B: those using only lifestyle modifications; Group C: those following only medication; Group D: those receiving no treatment (neither medication nor lifestyle modifications).

**Table 3:** Factors associated with treatment status after diagnosis of hypertension in young adults (reference: Group A).

Variables	Group B		Group C		Group D	
	OR	95% CI	OR	95% CI	OR	95% CI
Sex (man)	2.028	1.48–2.76	0.960	0.80–1.1	1.427	1.12–1.82
BMI	0.925	0.91–0.95	0.993	0.98–1.01	0.932	0.91–0.95
Smoking (past smoking)	0.581	0.43–0.79	1.057	0.83–1.35	0.811	0.61–1.08
Smoking (current smoking)	1.345	1.06–1.71	1.517	1.22–1.89	1.366	1.06–1.77
Alcohol drinking (≤ once/week)	1.690	1.21–2.36	0.793	0.63–0.99	1.654	1.14–2.39
Alcohol drinking (≥ twice/week)	1.907	1.28–2.84	1.427	1.07–1.91	2.484	1.59–3.87
Being aware of one's own blood pressure (yes)	0.980	0.27–0.54	0.699	0.53–0.93	0.194	0.15–0.26
Knowledge of stroke warning signs	0.870	0.79–0.96	0.920	0.85–0.99	0.912	0.83–1.00
Knowledge of myocardial infarction signs	0.932	0.84–1.03	0.964	0.89–1.04	0.779	0.71–0.86
Stress level	0.966	0.88–1.06	0.836	0.76–0.92	0.911	0.81–1.02
Addiction experience (yes)	1.057	0.82–1.37	0.826	0.65–1.05	1.630	1.27–2.90
Depressive experience (yes)	1.023	0.77–1.36	0.652	0.52–0.82	0.887	0.65–1.21
Education on how to manage hypertension (yes)	1.087	0.89–1.32	0.587	0.50–0.69	0.648	0.52–0.80
Social network level	0.965	0.94–0.99	0.975	0.96–0.99	0.934	0.91–0.96

*Note:* Group B: those using only lifestyle modifications; Group C: those following only medication; Group D: those receiving no treatment (neither medication nor lifestyle modifications). Reference: Group A, those undertaking both medication and lifestyle modifications (exercise and dietary adjustments). Variable reference: sex, woman; smoking, nonsmoking; alcohol drinking, nondrinking; being aware of one's own blood pressure, no; addiction experience, no; depressive experience, no; education on how to manage hypertension, no.

## Data Availability

The data that support the findings of this study are available from the KCHS, but restrictions apply to the availability of these data, which were used under approval for the current study and so are not publicly available. The data are, however, available from the authors upon reasonable request and with the permission of Korea Centers for Disease Control and Prevention (KCDC).
